# Combined immune checkpoint blockade for metastatic uveal melanoma: a retrospective, multi-center study

**DOI:** 10.1186/s40425-019-0800-0

**Published:** 2019-11-13

**Authors:** Markus V. Heppt, Teresa Amaral, Katharina C. Kähler, Lucie Heinzerling, Jessica C. Hassel, Markus Meissner, Nicole Kreuzberg, Carmen Loquai, Lydia Reinhardt, Jochen Utikal, Evelyn Dabrowski, Anja Gesierich, Claudia Pföhler, Patrick Terheyden, Kai-Martin Thoms, Lisa Zimmer, Thomas K. Eigentler, Michael C. Kirchberger, Henner M. Stege, Friedegund Meier, Max Schlaak, Carola Berking

**Affiliations:** 1 Department of Dermatology and Allergy, Munich University Hospital (LMU), Frauenlobstr. 9-11, 80337 Munich, Germany; 2 Department of Dermatology, University Hospital Erlangen, Friedrich-Alexander-University Erlangen-Nürnberg (FAU), Ulmenweg 18, 91054 Erlangen, Germany; 3 Department of Dermatology, Center for Dermatooncology, University Hospital Tübingen, Liebermeisterstr. 25, 72076 Tübingen, Germany; 4 Portuguese Air Force Health Care Direction, Lisbon, Portugal; 5 Department of Dermatology, University Hospital Schleswig-Holstein, Campus Kiel, Rosalind-Franklin-Str. 7, 24105 Kiel, Germany; 6 Skin Cancer Center, Department of Dermatology and National Center for Tumor Diseases (NCT), University Hospital Heidelberg, Im Neuenheimer Feld 460, 69120 Heidelberg, Germany; 7 Department of Dermatology, Venereology and Allergology, Goethe University, Theodor-Stern Kai 7, 60590 Frankfurt am Main, Germany; 8 Department of Dermatology and Venereology, Skin Cancer Center at the Center of Integrated Oncology (CIO) Köln Bonn, University Hospital of Cologne, Kerpenerstr. 62, 50937 Cologne, Germany; 9 Department of Dermatology, University Medical Center Mainz, Langenbeckstr. 1, 55131 Mainz, Germany; 10 Department of Dermatology, Skin Cancer Center, Medical Faculty and University Hospital Carl Gustav Carus, TU Dresden, Fetscherstr. 74, 01307 Dresden, Germany; 11 Skin Cancer Unit, German Cancer Research Center (DKFZ) and Department of Dermatology, Venereology and Allergology, University Medical Center Mannheim, Ruprecht-Karl University of Heidelberg, Theodor-Kutzer-Ufer 1-3, 68167 Mannheim, Germany; 12 Department of Dermatology, Klinikum Ludwigshafen, Bremserstr. 79, 67063 Ludwigshafen, Germany; 13 Department of Dermatology, University Hospital Würzburg, Josef-Schneider Straße 2, 97080 Würzburg, Germany; 14 Department of Dermatology, Saarland University Medical School, Kirrbergerstr, 66421 Homburg/Saar, Germany; 15 Department of Dermatology, University of Lübeck, Ratzeburger Allee 160, 23538 Lübeck, Germany; 16 Department of Dermatology, University Medical Center Göttingen, Robert-Koch-Str. 40, 37075 Göttingen, Germany; 17 Department of Dermatology, University Hospital, University Duisburg-Essen, Hufelandstr. 55, 45147 Essen, Germany

**Keywords:** Ipilimumab, Nivolumab, Combined immune checkpoint blockade, Uveal melanoma, Biomarker

## Abstract

**Background:**

Uveal melanoma (UM) is highly refractory to treatment with dismal prognosis in advanced stages. The value of the combined checkpoint blockade with CTLA-4 and PD-1 inhibition in metastatic UM is currently unclear.

**Methods:**

Patients with metastatic or unresectable UM treated with ipilimumab in combination with a PD-1 inhibitor were collected from 16 German skin cancer centers. Patient records of 64 cases were analyzed for response, progression-free survival (PFS), overall survival (OS), and safety. Clinical parameters and serum biomarkers associated with OS and treatment response were determined with Cox regression modelling and logistic regression.

**Results:**

The best overall response rate to combined checkpoint blockade was 15.6% with 3.1 and 12.5% complete and partial response, respectively. The median duration of response was 25.5 months (range 9.0–65.0). Stable disease was achieved in 21.9%, resulting in a disease control rate of 37.5% with a median duration of the clinical benefit of 28.0 months (range 7.0–65.0). The median PFS was 3.0 months (95% CI 2.4–3.6). The median OS was estimated to 16.1 months (95% CI 12.9–19.3). Regarding safety, 39.1% of treated patients experienced a severe, treatment-related adverse event according to the CTCAE criteria (grade 3: 37.5%; grade 4: 1.6%). The most common toxicities were colitis (20.3%), hepatitis (20.3%), thyreoiditis (15.6%), and hypophysitis (7.8%). A poor ECOG performance status was an independent risk factor for decreased OS (*p* = 0.007).

**Conclusions:**

The tolerability of the combined checkpoint blockade in UM may possibly be better than in trials on cutaneous melanoma. This study implies that combined checkpoint blockade represents the hitherto most effective treatment option available for metastatic UM available outside of clinical trials.

## Background

Uveal melanoma (UM) is a malignant tumor of the eye that originates from the pigment cells of the choroid layer or the ciliary body which is clinically and biologically distinct from cutaneous melanoma. Although the incidence is much lower than that of cutaneous melanoma, UM belongs to the most common malignant intraocular tumors in adults [1]. In approximately 50% of all cases, patients develop distant metastasis during the course of the disease, which affects predominantly the liver. Clinical risk factors for metastases are posterior localization in the eye, tumor size of more than 10 mm, and presence of vascular loops. Molecular biomarkers associated with a higher risk of metastasis are monosomy 3 or genomic alterations of BAP-1 [2]. Once distant metastases have occurred, the prognosis is dismal with an average survival time of approximately 1 year across all therapeutic regimens [3].

Patients with metastatic UM have so far benefited little or not at all from the treatment innovations achieved in cutaneous melanoma in recent years. Neither targeted therapy with MEK inhibitors nor checkpoint blockade with ipilimumab or PD-1 inhibitors as monotherapy was able to significantly improve the prognosis of patients with UM [4, 5]. The response rates were consistently in the single-digit percentage range in a panel of previous studies [6–9]. In cutaneous melanoma, combined checkpoint blockade with ipilimumab and nivolumab revealed response rates and survival outcomes superior to PD-1 inhibitor monotherapy, albeit at the cost of high immune-related toxicity [10]. However, the significance of combined checkpoint blockade in UM is unclear and has only been investigated in case reports and small case series [6, 11, 12]. In this study, we evaluate the clinical course of 64 patients with metastatic UM who received combined checkpoint blockade. We report clinical outcomes with respect to response, survival, and adverse events (AE). Furthermore, clinical and laboratory parameters were investigated which may have prognostic value in UM patients treated with checkpoint blockade.

## Patients and methods

### Patient population and study approval

This study was designed as a retrospective multi-center explorative analysis. Patients were included if they had a diagnosis of stage IV UM and received combined checkpoint blockade of ipilimumab with a PD-1 inhibitor in any treatment line. A follow-up period of at least 3 months was required. The clinical data of 64 patients from 16 German skin cancer centers who met the inclusion criteria were investigated. The cases were collected from June 23, 2018 to October 4, 2019. Clinical data and the treatment outcomes of interest were extracted from the original patient records and merged into a central database prior to analysis. This study was approved by the institutional review board of the medical faculty of the Munich University Hospital (approval number 413–16 UE) and was conducted in accordance with the principles of the Helsinki Declaration in its current version.

### Data collection and treatment outcomes

The clinical data recorded at baseline prior to immunotherapy comprised demographics with Eastern Cooperative Oncology Group (ECOG) performance status, available information on the genotype, sites of metastasis, number of organ systems affected by metastases, and previous antineoplastic therapies. As potential serum biomarkers, lactate dehydrogenase (LDH), C-reactive protein (CRP), and the relative counts of lymphocytes (RLC), neutrophils (RNC), and eosinophils (REC) were specifically collected from patient charts and analyzed for their prognostic value [13, 14].

Combined checkpoint blockade was carried out using different treatment schedules (Table 1). Ipilimumab was given at either 3 mg/kg or 1 mg/kg body weight for up to 4 treatment cycles. Nivolumab was applied at 1 mg/kg together with ipilimumab, followed by 3 mg/kg every 2 weeks (Q2W) as maintenance therapy. Treatment with pembrolizumab was applied every 3 weeks (Q3W) at 2 mg/kg. Patients were treated until disease progression or until the development of unacceptable toxicity. AE were retrospectively graded by the site investigators based on the patient records and clinical outcomes according to the Common Terminology Criteria for Adverse Events (CTCAE) v5.0 published by the National Institutes of Health in 2017. Immune-related adverse events were managed according to pertinent guidelines and algorithms that were previously published [15, 16]. Besides, fatal adverse events and events leading to permanent discontinuation of treatment were specifically recorded and evaluated. The best radiologic response to treatment was assessed by the site investigators and indicated as complete response, partial response, stable disease, or progressive disease based on the RECIST criteria version 1.1 [17]. Complete response and partial response were summarized as best overall response rate (ORR). Complete response, partial response, and stable disease were summarized as disease control rate (DCR).

**Table 1:** Baseline characteristics of the patient population

	Patient population *n* = 64 (100%)
Gender	
Male	33 (51.6)
Female	31 (48.4)
Age	
< 60 years	28 (43.8)
≥ 60 years	36 (56.2)
GNAQ	
Mutated	8 (12.5)
Wildtype	8 (12.5)
Unknown	48 (75.0)
GNA11	
Mutated	10 (15.6)
Wildtype	5 (7.8)
Unknown	49 (76.6)
ECOG status	
0	49 (76.6)
1	11 (17.2)
2	1 (1.6)
3	1 (1.6)
Unknown	2 (3.1)
Serum LDH	
Normal	28 (43.8)
Elevated (>ULN)	33 (51.6)
Unknown	3 (4.7)
Previous systemic therapies	
0	50 (78.1)
1	12 (18.8)
≥ 2	2 (3.1)
Previous ipilimumab monotherapy	2 (3.1)
Previous PD-1 inhibitor monotherapy	12 (18.8)
Liver-directed therapies	
0	33 (51.6)
1	30 (46.9)
≥ 2	1 (1.6)
Metastatic sites^a^	
Liver	58 (90.6)
Lung	23 (35.9)
Bone	17 (26.6)
Lymph nodes	12 (18.8)
CNS	4 (6.3)
Treatment regimen	
Ipilimumab 3 mg/kg + nivolumab 1 mg/kg Q3W, followed by nivolumab 3 mg/kg Q2W	59 (92.2%)
Ipilimumab 1 mg/kg + pembrolizumab 2 mg/kg Q3W, followed by pembrolizumab 2 mg/kg Q3W	5 (7.8%)

^a^Multiple metastatic sites per patient were possible (values do not sum up to 100%); *abbreviations*: *CNS* Central nervous system, *Q2W* Every two weeks, *Q3W* Every three weeks

### Statistical analyses

Overall survival (OS) and progression-free survival (PFS) were calculated as the time from the initiation of the first cycle of combined checkpoint blockade until melanoma-specific or treatment-related death and disease progression, respectively. Time-to-event analyses were calculated where death or progression were considered as events. If neither occurred or if patients were lost to follow-up, the date of the last documented presentation was used as a censored observation. The survival and progression probabilities were indicated with the Kaplan-Meier method for censored failure time data assuming proportional hazards. The survival curves were compared with the log-rank test [6]. The duration of the clinical response and clinical benefit was defined as time from treatment initiation to progressive disease if a response or stable disease was achieved, respectively. The time to response was defined as time from treatment start until a response was evident radiologically.

Cox proportional hazards regression modelling was applied to investigate the relationship of clinical risk factors and serum biomarkers with OS. Cox regression was performed as a univariate and multivariate analysis in a stepwise approach [6]. Imputation of missing data was not allowed and patients with missing values of a given parameter were excluded from the analysis. Hazard ratios (HR) with 95% confidence intervals (CI) were calculated to quantify the impact on survival. *P*-values were calculated based on Wald statistics [6]. The association of treatment response as a categorical variable with clinical characteristics or serum biomarkers was investigated with the Chi-square test and logistic regression, as appropriate. In all cases, two-tailed *p*-values were calculated and considered significant with values *p* < 0.05. All analyses were carried out with SPSS statistics version 23.0 (IBM) or GraphPad Prism version 5.01 (GraphPad Software).

## Results

A total of 64 (100%) patients with metastatic UM were included. Fifty patients (78.1%) were naïve to systemic treatment and received combined checkpoint blockade as first-line systemic therapy. Regarding genotype, the presence of monosomy 3 as risk factor was specifically investigated in 7 patients and identified in 2 of them. BRAF, NRAS and KIT were analyzed and reportedly wildtype as expected in 30, 22, and 20 patients, respectively. Mutations and inactivations of MBD4 which were previously linked to a hypermutator profile with high sensitivity to PD-1 inhibition were not investigated in any case [18, 19].

Previous ipilimumab and PD-1 inhibitor monotherapy were applied in 2 (3.1%) and 12 (18.8%) cases, respectively. Both patients treated with ipilimumab before showed PD. Specifically, 4 patients (6.3%) had received nivolumab and 8 (12.5%) pembrolizumab before. In 4 cases, SD was achieved while 8 patients showed PD upon PD-1 inhibitor monotherapy. The median duration of the clinical benefit was 6.5 months in the 4 patients with SD. Liver-directed therapies were reported in 31 patients (48.4%). Most patients had an ECOG status of 0 (*n* = 49, 76.6%). Serum LDH was elevated in 33 cases (51.6%) at baseline. Other baseline characteristics are listed in detail in Table 1. Ipilimumab plus nivolumab was given in 59 patients (92.2%), while 5 patients (7.8%) received ipilimumab plus pembrolizumab. The median number of treatment cycles was 3 (range 1–4) for the combination of ipilimumab with a PD-1 inhibitor in the induction phase, and 0 (range 0–27) for PD-1 inhibitor maintenance therapy in the overall population. A total of 19 patients (29.7%) received a PD-1 inhibitor maintenance therapy. Among these, the median number of PD-1 inhibitor cycles was 3 (range 1–27).

**Fig. 1:**
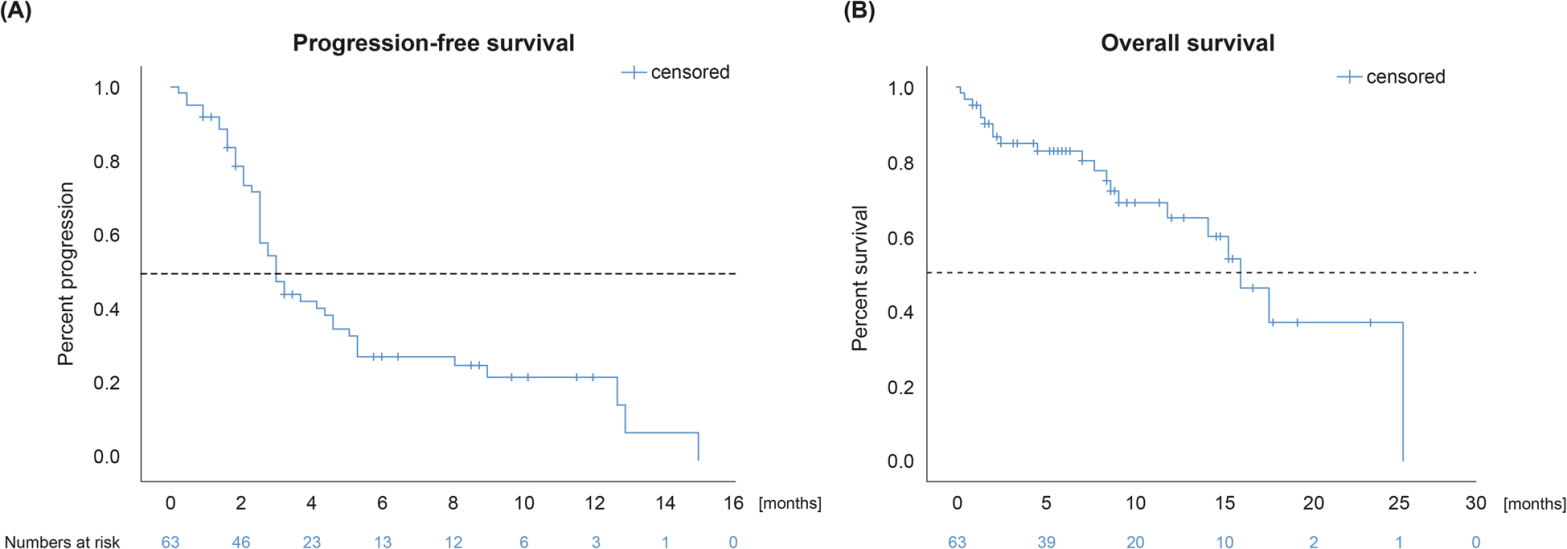
Kaplan-Meier estimates of the patient population for **a** progression-free survival (PFS) and **b** overall survival (OS). The median PFS and OS was estimated to 3.0 months (95% CI 2.4–3.6) and 16.1 months (95% CI 12.9–19.3), respectively. One patient was not included in the Kaplan-Meier analysis for PFS and OS due to missing data

The best ORR to combined checkpoint blockade was 15.6% (*n* = 10) relating to the entire population (4 patients were not evaluable for a radiologic response). Two patients achieved a complete response (3.1%) and 8 (12.5%) a partial response. The median duration of response was 25.5 months (range 9.0–65.0). Stable disease was achieved in further 14 cases (21.9%), resulting in a disease control rate of 37.5% with a median duration of the clinical benefit of 28.0 months (range 7.0–65.0) (Table 2). The median PFS was 3.0 months (95% CI 2.4–3.6). The median OS was estimated to 16.1 months (95% CI 12.9–19.3) with a median follow-up period of 9.2 months (95% CI 7.8–10.6) (Fig. 1).

**Table 2:** Best response rates to combined checkpoint blockade

	Cases (%)	Cumulative percentage (%)
Complete response	2 (3.1)	3.1
Partial response	8 (12.5)	15.6 (ORR)
Stable disease	14 (21.9)	37.5 (DCR)
Progressive disease	36 (56.3)	93.8
Unknown	4 (6.3)	100
Total	64 (100)	100

*Abbreviations*: *ORR* Objective response rate, *DCR* Disease control rate

The median time to response in patients with CR or PR after treatment initiation was 12 weeks (range 5–31). For the patients with SD, the median duration until the benefit was observed also amounted to 12 weeks (range 9–30). Interestingly, all 4 patients with SD after previous single PD-1 inhibitor blockade had PD to combined checkpoint blockade. Among the remaining 8 patients with PD after previous single PD-1 inhibitor blockade, one achieved a PR to combined checkpoint blockade. Thus, these data suggest that the effects of single and combined checkpoint blockade were observed independently from each other.

A total of 78 AE were reported in 39 patients. Thus, the majority of patients developed any treatment-related AE (60.9%). Of all events, 37 AE were graded as severe (grade 3 + 4). They were observed in 25 patients (39.1%; grade 3: 37.5%; grade 4: 1.6%). The treatment was discontinued in 25 cases (39.1%) due to unacceptable toxicity. However, no treatment-related deaths occurred during treatment or the observation period. The most common events were colitis (20.3%), hepatitis (20.3%), thyreoiditis (15.6%), hypophysitis (7.8%), fever (4.7%), and myalgia with myositis (4.7%). In all 5 cases with hypophysitis, the individual hormone axes including ACTH, cortisol, FSH, LH, TSH, and testosterone were investigated but not specifically graded. In 3 cases, the pituitary gland was enlarged in MRI examinations. All patients received systemic replacement of hydrocortisone. All AE are listed in Additional file 1.

**Fig. 2:**
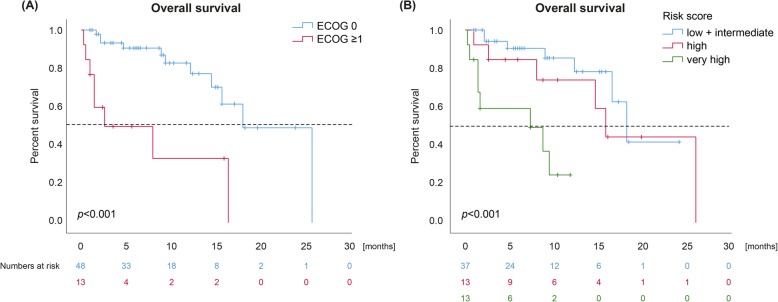
**a** Kaplan-Meier estimates for overall survival (OS) according to ECOG performance status. The median OS was 17.7 months (95% CI 13.1–22.3) for ECOG 0 versus 2.5 months (95% CI 0.0–9.6) for ECOG ≥1. Three patients were not included due to missing data. **b** Kaplan-Meier estimates for OS according to the prognostic score based on the serum parameters LDH, CRP, and REC. The groups with low and intermediate risk were pooled due to a small number of cases. The median OS was 17.7 months (95% CI 14.7–20.8) in the low plus intermediate group versus 15.4 months (95% CI 12.7–18.2) in the high risk group versus 7.1 months (95% CI 0.0–16.2) in the very high risk group. The *p*-values indicated were calculated with the log-rank test. One patient was not included due to missing data

In univariate Cox regression, ECOG status (*p* = 0.000096), the presence of bone metastasis (*p* = 0.011), and the best response to checkpoint blockade (*p* = 0.002) were significantly associated with OS (Additional file 2). The risk factors ECOG status, serum LDH, serum levels of CRP, and presence of bone metastasis were further integrated into a multivariate Cox regression model. Of these factors, a significant association with OS was confirmed for ECOG status (*p* = 0.007) only (Table 3, Fig. 2a).

**Table 3:** Multivariate Cox regression analysis of clinical parameters and serum biomarkers

Parameter	Category	HR (95% CI)	*P*-value
ECOG status	n.a. (ordinal)	3.19 (1.36–7.47)	0.007*
LDH	normal	1	0.428
	elevated (>ULN)	1.83 (0.41–8.08)	
CRP	normal	1	0.534
	elevated (>ULN)	1.73 (0.31–9.74)	
Bone metastasis	no	1	0.331
	yes	2.02 (0.49–8.27)	

Four parameters were included in the multivariate Cox regression analysis. Of these factors, ECOG status was significantly associated with overall survival in this model. *Abbreviations*: *CI* Confidence interval, *n.a.* not applicable, *ULN* Institutional upper limit of normal, *LDH* Lactate dehydrogenase, *CRP* C-reactive protein; *p<0.05.

We recently identified a prognostic score of the serum biomarkers LDH, CRP, and relative eosinophil count (REC) in a cohort of 94 UM patients receiving PD-1 inhibitors [6]. The score assigns one risk point for each unfavorable factor, i.e., elevated LDH, elevated CRP, and a REC < 1.5%, defining four distinct prognostic groups (low, intermediate, high, and very high risk). Each patient receiving combined checkpoint blockade was assigned to a risk group and the score was validated with Kaplan-Meier estimates. Due to a small sample size, patients with low and intermediate risk were pooled. The risk groups showed significantly different survival probabilities (*p* = 0.000005). The median survival times were superior for the low plus intermediate group (17.7 months, 95% CI 14.7–20.8) compared to the high (15.4 months, 95% CI 12.7–18.2) and very high risk group (7.1 months, 95% CI 0.0–16.2) (Fig. 2b). However, the score neither correlated with the response rate (*p* = 0.609) nor with the DCR (*p* = 0.446), suggesting that it was generally prognostic but not specifically predictive for the response to combined checkpoint blockade.

Subgroup analysis were performed for patients with metastasis to the central nervous system (CNS) at treatment initiation and for the treatment responders. Four patients showed an involvement of the CNS. Two of them had neurological symptoms. Two patients achieved SD, 2 showed PD. The median PFS for the CNS subgroup was 3.0 months (95% CI 0.0–6.1) while the median OS was not reached. In contrast, none of the treatment responders (CR or PR) had CNS involvement when the treatment was initiated (Table 4). The median time from detection of the primary tumor to metastatic disease was 43 months among the responders. Data on the assessment of the risk of metastasis formation of the primary tumors were sparse, as e.g. the presence of monosomy 3 or the MBD4 status was not investigated in any of the responders.

**Table 4:** Characterization of the responders to combined checkpoint blockade (*n* = 10)

Response	Duration of response (months)	Time to response (weeks)	Treatment cycles (induction + maintenance)	Gender	Time to metastasis from primary tumor (months)	Age at treatment onset (years)	Available molecular/ genetic analysis	ECOG	LDH	CRP	Risk score	Previous systemic treatment	Previous liver-directed treatment	Sites of metastasis	AE ≥ grade 3 (CTCAE v5.0)
PR	14	5	4 + 0	female	9	46	mutated: GNAQ (Q209P)	0	normal	normal	low	nivolumab (PD)	TACE	liver	yes (colitis)
PR	9	10	1 + 0	female	92	54	mutated: GNAQ (Q209P), ALK, MET; wildtype: BRAF, NRAS, GNA11, BAP1	0	normal	normal	low	none	none	lung	yes (colitis)
PR	23	10	4 + 0	female	33	77	mutated: GNA11	0	elevated	unknown	low	none	chemo-saturation	liver, mesenteric fat tissue	yes (Guillain-Barré syndrome)
PR	55	13	3 + 0	male	408	67	mutated: GNAQ	0	normal	elevated	high	none	surgery	liver, nodal	yes (colitis, hypophysitis)
PR	65	19	4 + 0	female	168	60	mutated: GNAQ; wildtype: BRAF, NRAS, KIT, GNA11	0	elevated	normal	high	none	TACE	liver, lung, ovarial, cervix, omentum	no
CR	53	12	4 + 5	male	unknown	59	unknown	0	unknown	unknown	low	none	surgery	lung	no
CR	50	12	4 + 16	male	unknown	45	unknown	0	elevated	elevated	very high	none	none	liver, bone, pelvic	no
PR	26	13	3 + 0	female	14	67	unknown	0	elevated	normal	intermediate	none	SIRT	liver, lung	yes (uveitis)
PR	25	14	4 + 1	female	30	56	wildtype: BRAF, NRAS, KIT; expression PD-L1 20%; polysomia of chromosome 12	0	elevated	elevated	high	none	none	liver, lung	no
PR	11	31	4 + 27	female	53	73	wildtype: BRAF, KIT, KRAS, NRAS, NF1, CDKN2A, CDK4	0	elevated	unknown	intermediate	none	chemo-saturation	liver, bone, nodal, renal	no

*Abbreviations*: *CR* Complete response, *PR* Partial response, *ECOG* Eastern Cooperative Oncology Group, *LDH* Lactate dehydrogenase, *CRP* C-reactive protein, *TACE* Transarterial chemoembolization, *SIRT* Selective internal radiation therapy, *AE* Adverse event(s), *CTCAE* Common Terminology Criteria for Adverse Events

## Discussion

Here, we present a comparatively large cohort of patients with metastatic UM who were treated with combined checkpoint blockade. We detected a 15.6% ORR, with a 3.1% complete and 12.5% partial response rate. This response rate is in line with our previous report showing 16% ORR, although only 12 patients were evaluable for their radiologic response and the follow-up time was short [6]. Another case series was recently published from a single-center experience where 2 out of 8 patients treated with nivolumab and ipilimumab had a partial response [11]. Other preliminary data on the efficacy of the combined checkpoint blockade have been proposed as conference abstracts, but appear preliminary to date. Najjar et al. reported results from a multi-center, retrospective analysis in 66 patients from 11 U.S. centers, revealing an ORR of 13% and a DCR of 31% [20]. In addition to these estimates in a real-world setting, prospective trials are currently underway. A preliminary analysis of the Spanish phase II trial GEM1402 (NCT02626962) showed an ORR of 12% and disease stabilization in 52% of cases [21]. Another phase II trial is currently ongoing in the U.S. in 30 patients with UM (NCT01585194). A recently presented interim analysis revealed an ORR of 17% and disease control in 50% [22]. Thus, we conclude that the ORR of 15.6% identified in this population is a solid estimate for the efficacy of combined checkpoint blockade in UM and a good indicator of what we can expect from the final analyses of the prospective trials. This regimen appears to be significantly superior compared to the sobering efficacy values observed with ipilimumab and PD-1 inhibitor monotherapy [6–9, 23–26]. Considering the data available so far, we conclude that the increase of ORR of the combined blockade versus PD-1 inhibition alone amounts to approximately 10%. Further evidence for a better efficacy of the combined regimen is supported by the observation of complete responders, albeit to a small extent. This is notable as UM is considered a “cold” tumor due to a low mutational burden and a unique immunosuppressive tumor microenvironment [27–29]. Further research is urgently needed to identify the radiologic, immunologic, and molecular determinants for treatment response in this small subset of patients. Regarding safety, the rate of severe AE was lower compared to the events reported in the pivotal trial in cutaneous melanoma (CheckMate-067) [30]. In particular, the occurrence of potentially life-threatening grade 4 AE was surprisingly low, suggesting that the regimen may be better tolerated in UM. However, it is also conceivable that the retrospective design and the small number of cases of this study causes an underreporting of AE.

Among clinical parameters and serum biomarkers, only the ECOG performance status was a consistent prognostic factor in multivariate analysis. Other parameters such as serum LDH, CRP, and the REC showed a significant association neither with OS nor with the treatment response when they were considered as single factors. However, when integrated into a prognostic score, they were useful for risk stratification and discriminated groups with distinct survival probabilities. Thus, the risk score identified previously in a distinct cohort was successfully validated in this population [6]. As there was a significant association neither with the ORR nor the DCR, we conclude that the score is generally prognostic but not specifically predictive for the response to checkpoint blockade.

The major limitations of this study are its retrospective design and the lack of a control group. When compared to historical controls, the median OS of 16.1 months is superior to survival estimates from other studies. Recently, the median OS benchmark for metastatic UM was identified as 10.2 months in a meta-analysis on individual data from 912 patients pooled from 29 trials [31]. Another analysis on individual-level data from 2494 patients proposed a median OS of 1.07 years across all treatment modalities. In this context, the OS observed in our cohort treated with combined checkpoint blockade appears more favorable, although external cohorts should be interpreted with caution and the comparison may be subject to significant confounding. A further limitation comes from the paucity of molecular and genetic analysis on the primary and metastatic tumors which are urgently needed to better characterize and understand the pattern of treatment response in UM.

## Conclusions

Altogether, our study implies that combined checkpoint blockade represents the hitherto most effective treatment option available for metastatic UM available in routine care outside of clinical trials. Based on our analysis and preliminary data from others, we hypothesize that the ORR achieved with combined checkpoint blockade will be 15–17%. Future trials are warranted to identify specific biomarkers for treatment response.

## Supplementary information

**Additional file 1.** Adverse events of combined checkpoint blockade according to frequency.

**Additional file 2.** Univariate Cox regression analysis of clinical and laboratory parameters.

## Data Availability

The datasets analysed during the current study are available from the corresponding author on reasonable request.

## Abbreviations

AE: Adverse event
CI: Confidence interval
CRP: C-reactive protein
CTCAE: Common Terminology Criteria for Adverse Events
DCR: Disease control rate
ECOG: Eastern Cooperative Oncology Group
HR: Hazard ratio
LDH: Lactate dehydrogenase
ORR: Overall response rate
OS: Overall survival
PFS: Progression-free survival
Q2W: Every two weeks
Q3W: Every three weeks
REC: Relative eosinophil count
RLC: Relative lymphocyte count
RNC: Relative neutrophil count
UM: Uveal melanoma
