# Supplementary material for: Combined immune checkpoint blockade for metastatic uveal melanoma: a retrospective, multi-center study
Source: J Immunother Cancer. 2019 Nov 13;7:299. doi: 10.1186/s40425-019-0800-0 (PMC6854774; doi:10.1186/s40425-019-0800-0)
Supplement: Supplementary file 1 — Additional file 1. Adverse events of combined checkpoint blockade according to frequency. [file 40425_2019_800_MOESM1_ESM.docx]

**Additional file 1: Adverse events of combined checkpoint blockade according to frequency**

| **Event** | **grade 1-2**  n (%) | **grade 3-4**  n (%) | **total**  n (%) |
| --- | --- | --- | --- |
| Colitis / Diarrhea | 3 (4.7) | 10 (15.6) | 13 (20.3) |
| Hepatitis | 5 (7.8) | 8 (12.5) | 13 (20.3) |
| Thyreoiditis | 9 (14.1) | 1 (1.6) | 10 (15.6) |
| Exanthema | 5 (7.8) | 0 (0.0) | 5 (7.8) |
| Hypophysitis | 0 (0.0) | 5 (7.8) | 5 (7.8) |
| Fever / Night sweats | 3 (4.7) | 0 (0.0) | 3 (4.7) |
| Myalgia / Myositis | 3 (4.7) | 0 (0.0) | 3 (4.7) |
| Diabetes mellitus / Hyperglycemia | 1 (1.6) | 1 (1.6) | 2 (3.1) |
| Fatigue | 0 (0.0) | 2 (3.1) | 2 (3.1) |
| Uveitis / Iritis | 1 (1.6) | 1 (1.6) | 2 (3.1) |
| Nephritis | 1 (1.6) | 1 (1.6) | 2 (3.1) |
| Pneumonitis | 1 (1.6) | 1 (1.6) | 2 (3.1) |
| Polyneuropathy | 0 (0.0) | 2 (3.1) | 2 (3.1) |
| Arthritis | 1 (1.6) | 0 (0.0) | 1 (1.6) |
| Cough | 1 (1.6) | 0 (0.0) | 1 (1.6) |
| Dysgeusia | 1 (1.6) | 0 (0.0) | 1 (1.6) |
| Elevated serum lipase | 0 (0.0) | 1 (1.6) | 1 (1.6) |
| Gastritis | 0 (0.0) | 1 (1.6) | 1 (1.6) |
| Guillain-Barré syndrome | 0 (0.0) | 1 (1.6) | 1 (1.6) |
| Hyperhidrosis | 1 (1.6) | 0 (0.0) | 1 (1.6) |
| Infusion reaction | 0 (0.0) | 1 (1.6) | 1 (1.6) |
| Lichen planus | 1 (1.6) | 0 (0.0) | 1 (1.6) |
| Myocarditis | 0 (0.0) | 1 (1.6) | 1 (1.6) |
| Pancreatitis | 0 (0.0) | 1 (1.6) | 1 (1.6) |
| Pruritus | 1 (1.6) | 0 (0.0) | 1 (1.6) |
| Sialadenitis | 1 (1.6) | 0 (0.0) | 1 (1.6) |
| Vitiligo | 1 (1.6) | 0 (0.0) | 1 (1.6) |
