# Supplementary material for: Combined immune checkpoint blockade for metastatic uveal melanoma: a retrospective, multi-center study
Source: J Immunother Cancer. 2019 Nov 13;7:299. doi: 10.1186/s40425-019-0800-0 (PMC6854774; doi:10.1186/s40425-019-0800-0)
Supplement: Supplementary file 2 — Additional file 2. Univariate Cox regression analysis of clinical and laboratory parameters. [file 40425_2019_800_MOESM2_ESM.docx]

**Additional file 2: Univariate Cox regression analysis of clinical and laboratory parameters**

| **No.** | **Factor** | **Hazard Ratio** | **95% CI** | **p-value** |
| --- | --- | --- | --- | --- |
| 1 | Gender | 0.923 | 0.376-2.263 | 0.860 |
| 2 | Age | 1.040 | 0.997-1.086 | 0.071 |
| 3 | GNAQ mut. | 1.074 | 0.582-1.982 | 0.818 |
| 4 | GNA11 mut. | 0.774 | 0.392-1.528 | 0.461 |
| **5** | **ECOG status** | **4.636** | **2.340-9.185** | **0.000*** |
| **6** | **LDH elevated** | **2.319** | **0.878-6.120** | **0.089** |
| **7** | **CRP elevated** | **4.352** | **0.975-19.420** | **0.054** |
| 8 | REC≥1.5% | 1.931 | 0.778-4.792 | 0.156 |
| 9 | RLC≥17.5% | 1.515 | 0.606-3.789 | 0.374 |
| 10 | RNC≥61.5% | 1.248 | 0.405-3.849 | 0.700 |
| 11 | Previous systemic therapies | 1.178 | 0.633-2.194 | 0.605 |
| 13 | Liver-directed therapies | 0.499 | 0.197-1.264 | 0.143 |
| 14 | Liver metastases | 1.900 | 0.253-14.299 | 0.533 |
| 15 | Lung metastases | 1.841 | 0.765-4.433 | 0.173 |
| **16** | **Bone metastases** | **3.163** | **1.303-7.678** | **0.011*** |
| 17 | Nodal metastases | 0.369 | 0.084-1.609 | 0.184 |
| 18 | CNS metastases | 1.229 | 0.161-9.375 | 0.843 |
| 19 | Number of metastatic sites | 1.141 | 0.806-1.614 | 0.457 |
| 20 | Any AE | 0.444 | 0.183-1.075 | 0.072 |
| 21 | Severe AE | 0.603 | 0.231-1.574 | 0.603 |
| 22 | Best response | 4.817 | 1.748-13.275 | 0.002* |

Factors which were further included in a multivariate Cox regression model are highlighted in bold; *p<0.05. Abbreviations: ECOG = Eastern Cooperative Oncology Group, REC = relative eosinophil count, RLC = relative lymphocyte count, RNC = relative neutrophil count; LDH = lactate dehydrogenase; CRP = C-reactive protein; CNS = central nervous system; CI = confidence interval; AE = adverse event.
